# Hump-Nosed Pit Viper (*Hypnale hypnale*) Venom-Induced Irreversible Red Blood Cell Aggregation, Inhibition by Monovalent Anti-Venom and N-Acetylcysteine

**DOI:** 10.3390/cells13120994

**Published:** 2024-06-07

**Authors:** Vaddaragudisalu D. Sandesha, Puttaswamy Naveen, Kurnegala Manikanta, Shanmuga S. Mahalingam, Kesturu S. Girish, Kempaiah Kemparaju

**Affiliations:** 1 Department of Studies in Biochemistry, University of Mysore, Manasagangotri, Mysuru 570006, Karnataka, India; sandeshpandiyan25@gmail.com (V.D.S.); naveenputtaswamy17@gmail.com (P.N.); manikantaakul5@gmail.com (K.M.); 2 Department of Biological Sciences, School of Dental Medicine, Case Western Reserve University, Cleveland, OH 44106, USA; sxm1505@case.edu; 3 Department of Studies and Research in Biochemistry, Tumkur University, Tumakuru 572103, Karnataka, India

**Keywords:** *H. hypnale* venom, RBC aggregation, hemotoxic, N-acetylcysteine, pro-coagulant

## Abstract

Envenomation by the *Hypnale hypnale* in the Western Ghats of India (particularly in the Malabar region of Kerala) and the subcontinent island nation of Sri Lanka is known to inflict devastating mortality and morbidity. Currently, *H. hypnale* bites in India are devoid of anti-venom regimens. A detailed characterization of the venom is essential to stress the need for therapeutic anti-venom. Notably, the deleterious effects of this venom on human blood cells have largely remained less explored. Therefore, in continuation of our previous study, in the present study, we envisioned investigating the effect of venom on the morphological and physiological properties of red blood cells (RBCs). The venom readily induced deleterious morphological changes and, finally, the aggregation of washed RBCs. The aggregation process was independent of the ROS and the intracellular Ca^2+^ ion concentration. Confocal and scanning electron microscopy (SEM) images revealed the loss of biconcave morphology and massive cytoskeletal disarray. Crenation or serrated plasma membrane projections were evenly distributed on the surface of the RBCs. The venom did not cause the formation of methemoglobin in washed RBCs but was significantly induced in whole blood. Venom did not affect glucose uptake and Na^+^/K^+^ -ATPase activity but inhibited glucose 6 phosphate dehydrogenase activity and decreased the fluidity of the plasma membrane. Venom-induced RBC aggregates exhibited pro-coagulant activity but without affecting platelet aggregation. In pre-incubation or co-treatment studies, none of the bioactive compounds, such as melatonin, curcumin, fisetin, berberine, and quercetin, sugars such as mannose and galactose, and therapeutic polyvalent anti-venoms (Bharat and VINS) were inhibited, whereas only N-acetylcysteine and *H. hypnale* monovalent anti-venom could inhibit venom-induced deleterious morphological changes and aggregation of RBCs. In post-treatment studies, paradoxically, none of the bioactives and anti-venoms, including N-acetylcysteine and *H. hypnale* monovalent anti-venom, reversed the venom-induced RBC aggregates.

## 1. Introduction 

In tropical countries in Asia, Africa, and Latin America, snakebites are a noteworthy occupational hazard that demands serious attention. In 2017, the World Health Organization (WHO) reported that each year, an estimated 5.4 million individuals are bitten by snakes and, among them, roughly 2.7 million develop clinical symptoms that require medical attention. Of these, 0.14 million die, while 0.4 million are permanently disabled [1]. In considering this macabre situation, the WHO recognized snakebites as a neglected tropical disease (NTD) in June 2017 [2]. Strikingly, approximately 58,000 people die each year from snakebite-related ailments in India, largely driven by the “Big Four” snakes: spectacled cobra (*Naja naja*), common krait (*Bungarus caeruleus*), saw-scaled viper (*Echis carinatus*), and Russell’s viper (*Daboia russelii*) [2,3,4]. The principal toxins in snake venom are phospholipase A_2_ (PLA_2_), serine proteinases (SVSPs), metalloproteinases (SVMPs), and three-finger toxins (3FTx). These toxins result in devastating systemic toxicities, such as neurotoxicity, cardiotoxicity, bleeding, coagulopathy, thrombosis, and acute kidney injury; however, they can also cause debilitating local toxicities, such as blisters, edema, hemorrhage, and persistent tissue necrosis, which can result in permanent deformities [5,6,7]. In addition to the “Big Four”, *Hypnale hypnale* is a venomous pit viper species endemic to the Western Ghats of India (Malabar region of Kerala) and the adjacent island nation of Sri Lanka [8]. Three species of *Hypnale* are known, but only *H. hypnale* (*Hh*) is distributed in India, while, in addition, *H. zara* and *H. nepa* are endemic to Sri Lanka [9]. However, there has been an alarming increase in the incidence of *Hh* bites in the Malabar region of Kerala, which has led to life-threatening and fatal complications. For several years, the underreporting of *Hh* envenomation in India was due to misidentification as either *D. russelii* or *E. carinatus* [8]. In the spirit of trying to increase public awareness regarding *Hh* bite and their management, our research has recently focused on comparing the biochemical, pathological, and immunological properties of Sri Lankan *Hh* venom (Indian *Hh* venom is not available) with the Indian *E. carinatus* and *D. russelii* venoms. We recently demonstrated that monovalent anti-venoms of *E. carinatus* and *D. russelii* did not cross-react with *Hh* venom; as a result, they were unable to neutralize its toxicities, whereas *Hh* monovalent anti-venom neutralized its venom effectively. Additionally, we showed that therapeutic polyvalent anti-venoms (Bharat and VINS) were ineffective in averting the toxicities of *Hh* venom, which is raised against the “Big Four” [10]. Unfortunately, *Hh* bites in India remain harmful without suitable anti-venom therapy.

In the recent past, various antioxidant molecules, such as melatonin [11], quercetin [12], curcumin [13], lupeol derivatives [14], crocin [15], N-acetylcysteine (NAC) [16], and N-acetylcysteine amide [17], have been extensively investigated for their potential to deter the systemic and local effects of snake venom. The natural antioxidant NAC is often used in the therapeutic management of psychiatric and neurological disorders, such as acetaminophen-induced toxicity, atrial fibrillation (Afib), and nephrotoxicity [18]. In 2022, three cases of venom-induced consumption coagulopathy (VICC), thrombotic microangiopathy, and acute renal damage from *Hh* snakebites were reported in Karnataka (Coastal Southwest India) [19]. Considering the increasing incidence of *Hh* envenomation, it is vital to explore the toxic effects of *Hh* venom on human blood cells, particularly red blood cells (RBCs), because they have not been systematically investigated. RBCs are the most abundant blood cells (5 million cells/μL), with a lifespan of approximately 120 days, and are the key players in the physiological gas exchange process [20]. Therefore, this study aimed to investigate the deleterious effects of *Hh* venom on RBCs and neutralization by anti-venoms, sugars, and bioactives.

## 2. Materials and Methods

### 2.1. Chemicals, Reagents, Anti-Venoms, and Snake Venom

Annexin V, Calcein-AM, Berberine, Complete, Mini, EDTA-free protease inhibitor cocktail, Dimethyl sulfoxide (DMSO), 2′,7′-dichlorodihydrofluorescein diacetate (H_2_DCFDA), Fisetin, Freund’s complete and incomplete adjuvants, Fura-2AM, Glutaraldehyde, HEPES (4-(2-hydroxyethyl)-1-piperazineethanesulfonic acid), Mannose, N-acetylcysteine (NAC), and Protein-A agarose were purchased from Sigma (New York, NY, USA). Alexa Fluor 488 Phalloidin was purchased from Thermo Fisher Scientific Inc. (Carlsbad, CA, USA). Commercial therapeutic polyvalent equine anti-snake venom serums were purchased from Bharat Serums and Vaccines, Ltd. (Navi Mumbai, Bharat), Mumbai, India (Batch number: A05320019, expiry date: 05/24), and VINS Bioproduct Ltd. (VINS), Hyderabad, India (Batch number 01AS21057, expiry date: 07/25). Thrombin was purchased from Chrono-log Corporation 2 W Park Road, Havertown, PA, USA. The Lactate Dehydrogenase (LDH) kit was purchased from Agappe Diagnostics, Ltd. (Ernakulam, India). The glucose test kit was purchased from Arkray Healthcare Pvt. Ltd. Surat, India. Adenosine-5-triphosphate disodium salt (ATP-Na_2_), Ammonium molybdate, Ascorbic acid, Citric acid, Curcumin, Dextrose, D-galactose, Ethylenediaminetetraacetic acid (EDTA), Glucose 6 phosphate, Melatonin, Magnesium chloride (MgCl_2_), Sodium chloride (NaCl), Disodium hydrogen phosphate (Na_2_HPO_4_), Potassium chloride (KCl), Potassium dihydrogen phosphate (KH_2_PO_4_), Quercetin, Sodium nitrite (NaNO_2_), Triton X-100, and Trisodium citrate, were purchased from Sisco Research Laboratory (Mumbai, India), and all other chemicals were of analytical grade. *Hypnale hypnale* venom (*Hh*v) was purchased from Latoxan Laboratory (lot. No. 317.081 & Product ID; L1602) France, as it is not marketed in India. It was purchased with permission from the Principal Chief Conservator of Forests (Wildlife) and the Chief Wildlife Warden, Karnataka State Forest Department, Govt. of Karnataka, India (Wildlife Permission No. PCCF(WL)/E2/CR-08/2019-20).

### 2.2. Ethics Statement

All the experiments were approved by the Institutional Human Ethical Committee (IHEC-UOM No. 70/Res/2020–21), University of Mysore, Mysuru, and conducted following ethical guidelines.

Animal experiments were approved by the Institutional Animal Ethical Committee (UOM/IAEC/04/2020), Department of Studies in Zoology, University of Mysore, Mysuru, and were conducted in accordance with the guidelines of the Committee for the Purpose of Control and Supervision of Experiments on Animals (CPCSEA).

### 2.3. Preparation of Hh Monovalent Anti-Venom and Affinity Purification of Immunoglobulin G Fraction

The immunization of rabbits and the purification of antibodies were performed as described by Shashidharamurthy et al. [21]. Briefly, *Hh*v (100 μg) was dissolved in 100 μL PBS (10 mM phosphate-buffered saline, pH 7.4), mixed thoroughly with an equal volume of Freund’s complete adjuvant, and injected intradermally (i.d.) at 4 different sites (50 μL for each site) in the back of 2-month-old female rabbits weighing about 1.5 to 2 Kgs (n = 3). Three booster doses of venom were administered at the same dosage, but with an equal volume of Freund’s incomplete adjuvant, at weekly intervals. Approximately 10 mL of blood was drawn from the marginal ear vein on the 9th day after the third booster dose and allowed to coagulate for 24 h at 8–10 °C to obtain anti-serum. About 6 mL of anti-serum was subjected to ammonium sulfate precipitation to obtain the crude immunoglobulin G fraction, which was subjected to protein-A agarose affinity column chromatography. The column was equilibrated with PBS and loaded with 5 mg of crude immunoglobulin G fraction in 2 mL of PBS. The elution was carried out using 0.2 M glycine-HCl buffer and pH 2.9. Aliquots and 1 mL was collected and pooled after reading the optical density at 280 nm and then neutralized using 1 M Tris–HCl buffer pH 8.0. The samples were further subjected to dialysis using a 3.4 kDa membrane against PBS for 24 h at 4 °C. Thus, the obtained monovalent anti-venom was designated *Hh* anti-venom (*Hh*AV) and used for the neutralization study.

### 2.4. Preparation of Bioactives, Sugars, and Anti-Venoms for Inhibition of RBCs Aggregation or Disaggregation Studies

Bioactives such as melatonin, curcumin, fisetin, berberine, and quercetin were dissolved independently in DMSO (0.1%), made into 10 mM stocks using PBS, and stored at −20 °C. NAC (10 mM) was prepared in phosphate-buffered saline (PBS). The mannose and galactose were dissolved in PBS and made into a stock at 10 mg/mL using PBS, then stored at 4 °C. The therapeutic polyvalent anti-venoms from Bharat and VINS were dissolved in sterile water according to the manufacturer’s instructions; *Hh*AV was dissolved in PBS, and protein estimation was performed to adjust the concentration of anti-venom. The protein concentration was estimated using Lowry’s method [22]. The prepared bioactives, sugars, and anti-venoms were used to inhibit venom-induced RBC aggregation in pre-incubation or co-treatment and to disaggregate the RBC aggregates in post-treatment studies in the respective experiments.

### 2.5. Isolation of Human Red Blood Cells (RBCs) 

Blood was drawn from non-smoking and non-medicated healthy human donors and instantly mixed with an acid citrate dextrose (ACD) anticoagulant (85 mM sodium citrate, 78 mM citric acid, and 111 mM D-glucose) at a ratio of 6:1 (blood−ACD, *v*/*v*) following centrifugation at 700× *g* for 15 min to separate the RBCs from the rest of the components. The supernatant was discarded, and the pellet was washed 3–4 times with PBS (1:1, *v*/*v*) at 200× *g* for 10 min. Washed RBCs were used in various cell-based assays [23].

### 2.6. Preparation of Platelet-Rich Plasma and Washed Platelets

For the preparation of platelet-rich plasma (PRP), the drawn blood was immediately mixed with an anticoagulant (3.2% trisodium citrate) at a ratio of 9:1 (blood−anticoagulant, *v*/*v*). Anticoagulated whole blood was centrifuged at 100× *g* for 16 min. The obtained blood supernatant (PRP) was then used for the platelet aggregation. For washed platelets, blood was immediately mixed with anticoagulant (ACD) at a ratio of 6:1 (blood−ACD, *v*/*v*). Anticoagulated whole blood was centrifuged at 100× *g* for 16 min to obtain platelet-rich plasma (PRP). After centrifugation at 500× *g*, the obtained platelet pellet was washed twice with CGS (123 mM NaCl, 33 mM D-glucose, 13 mM trisodium citrate, and pH 6.5) and re-suspended in HEPES-buffered saline (HBS, 2.5 mM HEPES, 15 mM NaCl, 2.5 mM KCl, 12 mM NaHCO_3_, 1 mM CaCl_2_, 1 mM MgCl_2_, 5.5 mM D-glucose, and pH 7.4). The suspended cells were counted using a Neubauer chamber and the cell count was adjusted to 5 × 10^6^ cells/mL [24]. 

### 2.7. Bright-Field Microscopic Images of RBCs Using Giemsa Stain 

Washed RBCs (0.1% hematocrit in PBS and 0.5 mL) were independently treated with increasing doses of *Hh*v (0–10 µg/mL) for 1 h at 37 °C. RBCs treated with PBS served as negative controls in all experiments. The cells were seeded in a 24-well culture plate on 12 mm sterile round coverslips. After treatment, the cells were fixed with 2.5% glutaraldehyde solution (in PBS) for 2 h at room temperature (RT). After fixation, cells were stained using 2.5% Giemsa stain (in PBS) for 30 min at RT followed by 2 washes with PBS. Further, coverslips were mounted using fluoromount on a clean glass slide and shade-dried. The cells were observed under the microscope using 40× magnification (QUASMO- PZQ106D fluorescence microscope, Quality Scientific & Mechanical Works, Ambala, India) [25]. For inhibition studies, the *Hh*v (10 µg/mL) was independently pre-incubated with various doses (0–1000 µg/mL) of anti-venoms (Bharat, VINS, and *Hh*AV) for 10 min at RT before treatment. Various doses (0–1000 µM) of bioactives such as melatonin, curcumin, fisetin, berberine, quercetin, and N-acetylcysteine, and various doses (0–500 µg/mL) of sugars, such as mannose and galactose, were independently treated with *Hh*v (10 µg/mL) for 10 min at RT before treatment. 

### 2.8. Methemoglobin Formation in Washed RBCs and Whole Blood

Methemoglobin (MetHb) formation in the whole blood was monitored as described by Benesch et al. [26] with slight modifications. Briefly, the anticoagulated blood (20 µL) was taken in a 96-well flat-bottom microtiter plate and the final volume was made up to 0.2 mL using PBSG (PBS containing 0.1% glucose). Diluted whole blood was treated independently with various doses of *Hh*v (0–100 µg/mL) for 16 h at 37 °C. For washed RBCs, 0.2 mL (0.5% hematocrit in PBSG) of cell suspension was treated independently with increasing doses of *Hh*v (0–100 µg/mL) for 16 h at 37 °C. After treatment, the cells were lysed with PBS containing 1% Triton X-100 (0.5:1, *v*/*v*), and the plates were read at 630 nm using a Tecan multi-mode plate reader (Infinite 200 Pro, Tecan, Grodig, Austria). Both washed RBCs and whole blood treated with NaNO_2_ (2 mM) served as the positive control while the PBS-treated samples served as the negative control. 

### 2.9. Estimation of Lactate Dehydrogenase (LDH) Release

The LDH released from washed RBCs in the presence of *Hh*v was estimated using the LDH kit according to the manufacturer’s protocol [25]. Briefly, washed RBCs (0.5% hematocrit in PBS) and (0.1 mL) were incubated with increasing doses of *Hh*v (0–100 μg/mL) for 1 h at 37 °C. Thereafter, samples were centrifuged at 1500× *g* for 10 min at 4 °C. Then, the obtained cell supernatant was used for the estimation of LDH release using the Agappe LDH assay kit. The PBS-treated cells served as the negative control and H_2_O_2_ (100 μM) treated cells served as the positive control. 

### 2.10. Estimation of Reactive Oxygen Species (ROS) and Intracellular Calcium (Ca^2+^) Ion Concentration

The total ROS was quantified fluorometrically in washed RBCs using DCFDA dye [27]. Briefly, the washed RBCs (0.2% hematocrit in PBS), 0.1 mL were added into the 96-well flat-bottom microtiter plate and treated independently with increasing doses of *Hh*v (0–100 μg/mL) for 1 h at 37 °C. After treatment, DCFDA was added to a final concentration of 10 μM and incubated for 30 min at RT to allow for the cleavage of DCFDA by esterases and further conversion into the fluorescent product dichloro-fluorescein. Then, fluorescent intensity was measured using a Tecan multi-mode plate reader (Infinite 200 Pro, Tecan, Grodig, Austria) with the excitation and emission wavelengths of 488 nm and 530 nm, respectively. For intracellular Ca^2+^ ion concentration, PBS and venom-treated cells were incubated with 2 μM Fura-2 AM for 30 min at RT. The Fura-2 AM absorption was determined by exciting the cells at 340 and 380 nm, and the resulting fluorescence was measured at 500 nm. Data were presented as absorption ratios (340/380 nm). H_2_O_2_ (100 μM) was used as the positive control and PBS-treated cells served as the negative control [28].

### 2.11. Measurement of Osmotic Fragility

RBC’s osmotic fragility was measured as the percentage of hemolysis induced by hypotonic solution. An osmolarity (which produces 50% of hemolysis) was calculated as described accordingly [29]. Briefly packed erythrocytes (50 μL) were independently pretreated with increasing doses of *Hh*v (0–100 μg/mL) for 10 min at RT. The PBS-treated samples served as control experiments. High glucose (HG), 50 mM served as positive control. After incubation, 1 mL of 0.55% NaCl was added to each tube and incubated for 30 min at RT. The samples were then subjected to centrifugation at 450× *g* for 10 min; supernatants were collected and hemolysis was then estimated by measuring the absorption of hemoglobin released at 540 nm using a UV spectrophotometer (Thermo Scientific, Waltham, MA, USA).

### 2.12. Determination of Glucose-6 Phosphate Dehydrogenase Activity

Glucose-6 phosphate dehydrogenase (G6PDH) activity was estimated by monitoring the increase in absorbance at 340 nm for 3 min due to NADP-dependent glucose 6-phosphate transformation [30]. Briefly, the washed RBCs (5% hematocrit in PBS) were treated with increasing doses of *Hh*v (0–100 μg/mL) for 1 h at 37 °C. The PBS-treated cells served as the negative control. HG (50 mM) served as the positive control. After incubation, the samples were subjected to centrifugation at 500× *g* for 10 min, supernatants were discarded, and pellets were lysed using 1 mM PBS. The lysates were used to determine G6PDH activity in the reaction mixture (1 mL) containing Tris-HCl buffer (50 mM, pH 7.5 containing 3.8 mM NADP^+^, 3.3 mM glucose-6-phosphate, and 6.3 mM MgCl_2_). The activity was expressed as an n mol NADPH formed/min/mg protein.

### 2.13. Measurement of Glucose Utilization

The glucose utilization levels were measured using a commercially available glucose test kit. Briefly, the washed RBCs (10% hematocrit in PBS) were supplemented with 5 mM glucose and treated with increasing doses of *Hh*v (0–100 μg/mL) for 1 h at 37 °C. The PBS-treated cells served as negative control. HG (50 mM) served as a positive control. After incubation, the concentration of glucose was measured at (0 h) and after 1 h of incubation by glucose oxidase reagent according to the manufacturer’s protocol. The absorbance was measured at 505 nm. The levels of glucose utilization were calculated by subtracting the glucose concentration at 1 h from the glucose concentration at 0 h using the equation below. The result was expressed as m mol/L/h [31].

Glucose utilization (m mol/L) = Glucose concentration at 0 h − Glucose concentration at 1 h.

### 2.14. Measurement of Na^+^/K^+^ -ATPase Activity

The Na^+^/K^+^ -ATPase activity was measured accordingly with minor modifications [32]. Briefly, the washed RBCs (10% hematocrit in PBS) were treated with various doses of *Hh*v (0–100 μg/mL) for 1 h at 37 °C. The PBS-treated cells served as negative control. HG (50 mM) served as a positive control. For the preparation of RBC ghost samples, after-venom treatment erythrocyte membranes were isolated following the described experimental protocol with slight modifications [33]. Briefly, after treatment with *Hh*v for 1 h at 37 °C, samples were subjected to centrifugation at 1000× *g* for 10 min and the supernatant was discarded. The cells were then lysed with 30 volumes of ice-cold lysis buffer (5 mM phosphate buffer pH 8 containing 1 mM EDTA along with protease inhibitor cocktail containing 10 μg/mL pepstatin A, 10 μg/mL leupeptin, 5 μg/mL aprotinin and 0.1 mM PMSF) for 20 min at 4 °C. RBC ghost samples were isolated, and their protein contents were determined by Lowry’s method [22]. The prepared RBC ghost samples were incubated with reaction buffer A containing 4 mM MgCl_2_, 3 mM ATP-Na_2_, and 50 mM Tris-HCl, pH 7.4, and buffer B containing 120 mM NaCl, 20 mM KCl, 4 mM MgCl_2_, 3 mM ATP-Na_2_, and 50 mM Tris-HCl, pH 7.4 at 37 °C for 1 h. After the incubation, the levels of phosphate (Pi) released from ATP-Na_2_ were measured accordingly with minor modifications [32]. The reaction mixture was incubated with ammonium molybdate (2.5%, *w*/*v*) at RT for 10 min. Then, ascorbic acid (2%, *w*/*v*) was added and kept at RT for 20 min for color development. The absorbance was measured at 725 nm. The levels of Pi released were calculated from a standard curve using KH_2_PO_4_. The Na^+^/K^+^ -ATPase activity was calculated using the equation below. The results were expressed as n mol Pi/mg protein/h.

Na^+^/K^+^ -ATPase activity = Pi in the reaction buffer B − Pi in the reaction buffer A

### 2.15. Flow Cytometric Analysis (FACS)

To estimate the phosphatidylserine (PS) externalization, the washed RBCs were treated with *Hh*v. Washed RBCs (0.2% hematocrit in PBS) were independently treated with increasing doses of *Hh*v (0–100 μg/mL) for 1 h at 37 °C. Cells treated with H_2_O_2_ (100 μM) served as a positive control and PBS-treated cells served as negative control. RBCs were further incubated with FITC-conjugated Annexin-V (0.5 μg/mL) for 30 min at RT. After incubation, the excess dye was removed by washing, followed by re-suspending of the cells with 0.2 mL of PBS. The Annexin-V absorption was analyzed by excitation and emission wavelengths of 496 nm and 516 nm, respectively, using a FACS Verse flow cytometer (BD Biosciences, San Jose, CA, USA) [34].

### 2.16. Effect of RBC Aggregates on Plasma Recalcification Time 

The plasma recalcification time was determined according to the method of Rosenberg et al. [35]. Briefly, freshly collected healthy human blood was mixed with 3.2% trisodium citrate in a ratio of 9:1 (*v*/*v*). The blood was centrifuged for 15 min at 500× *g*. The obtained supernatant was used as platelet-poor plasma (PPP) kept at 37 °C and used within 4 h from the drawing of blood. Washed RBCs (0.5% hematocrit in PBS) were treated with different doses of *Hh*v (0–10 μg/mL) and incubated for 1 h at 37 °C. The obtained respective RBC aggregates were washed (3 times) thoroughly with PBS and tested for the effect on plasma recalcification time. This assay mixture contains 0.2 mL PPP and respective RBC aggregates, pre-incubated for 1 min at RT in the presence of 10 mM Tris-HCl pH 7.4 (10 μL), and clotting was initiated by adding 0.25 M CaCl_2_. To determine the normal recalcification time, PPP (0.2 mL) was treated with PBS (10 μL), followed by a quick addition of 0.25 M CaCl_2_. For inhibition studies, the RBC aggregates obtained at 10 μg/mL venom were thoroughly washed with PBS and then used. RBC aggregates (20 μL) were independently pre-incubated with various doses (0–500 μg/mL) of anti-venoms (Bharat, VINS, and *Hh*AV) for 5 min at RT. This reaction mixture was added to 0.2 mL PPP and incubated for 1 min at RT. Clotting was initiated by adding 0.25 M CaCl_2_. In all the cases, clotting time was recorded in seconds against a light source. 

### 2.17. Effect of RBC Aggregates on Platelet Aggregation

The turbidimetric method of Born [36] was followed using a Chronolog dual-channel whole blood/optical Lumi aggregation system (Model-700). Aliquots of 250 µL PRP/washed platelets were independently taken in a siliconized glass cuvette, and the aggregation was initiated by adding 20 µL of aggregated RBCs. The aggregation was followed with constant stirring at 1200 rpm for 6 min against platelet-poor plasma (PPP). For washed platelets, the suspension buffer of 500 µL HBS was used. Thrombin 0.1 U was used as a positive control. 

### 2.18. Determination of Viability of RBCs Using Calcein-AM 

In regard to the washed RBCs (0.1% hematocrit in PBS and 0.5 mL) were independently treated with increasing doses of *Hh*v (0–10 µg/mL) for 1 h at 37 °C. For the confocal study, cells were seeded in a 24-well culture plate on 12 mm round coverslips. For spectroscopic analysis, 0.2% hematocrit (0.1 mL) was added to a 96-well flat-bottom microtiter plate. RBCs treated with H_2_O_2_ (100 μM) served as a positive control and PBS-treated cells served as a negative control. After treatment, cells were stained with 1 µM of calcein-AM fluorescent dye for 30 min at RT followed by 2 washes with PBS. The calcein-AM absorption was analyzed by excitation and emission wavelengths of 495 nm and 515 nm, respectively, using a Tecan multi-mode plate reader (Infinite 200 Pro, Tecan, Grodig, Austria). For fluorescence imaging, cells were stained with 1 µM of calcein-AM fluorescent dye for 30 min followed by washing (2 times) with PBS and fixed with 2.5% glutaraldehyde solution for 2 h at RT. After fixation, coverslips were mounted using a fluoromount on clean glass slides and dried under dark conditions [37]. The cells were observed under a confocal laser-scanning system using 63× oil magnification (Carl Zeiss confocal microscope LSM 710, ZEISS, Germany). Images and fluorescence intensities were processed by ZEN lite software (ZEN 2010; Ver.6.0, EMBL Heidelberg, Germany) from Zeiss microscopy (Heidelberg, Germany). For inhibition studies, the *Hh*v (10 µg/mL) was independently pre-incubated with various doses (0–1000 µg/mL) of anti-venoms (Bharat, VINS, and *Hh*AV) and N-acetylcysteine (0–500 µM) for 10 min at RT before the treatment. 

### 2.19. Cytoskeletal F-Actin Disorganization in RBCs

The washed RBCs (0.1% hematocrit in PBS and 0.5 mL) were independently incubated with increasing doses of *Hh*v (0–10 µg/mL) for 1 h at 37 °C. The cells were seeded in a 24-well culture plate on 12 mm sterile round coverslips. After treatment, cells were fixed with 2.5% glutaraldehyde for 2 h at RT. Then, cells were permeabilized with 0.1% triton-X-100 in PBS for 5 min followed by 2 washes using PBS. Further cells were stained with F-actin-specific phalloidin coupled with Alexa Fluor 488 (2 units/sample) at RT for 2 h in the dark. Confocal images were acquired with a confocal laser-scanning system using 63× oil magnification (Carl Zeiss confocal microscope LSM 710, ZEISS, Germany). Images and fluorescence intensities were processed by ZEN lite software (ZEN 2010; Ver.6.0, EMBL Heidelberg, Germany) from Zeiss microscopy [25]. For inhibition studies, the *Hh*v (10 µg/mL) was independently pre-incubated with various doses (0–1000 µg/mL) of anti-venoms (Bharat, VINS, and *Hh*AV) and NAC (0–500 µM) for 10 min at RT before the treatment. 

### 2.20. Scanning Electron Microscopy (SEM) of Hhv-Treated RBCs

In regard to the washed RBCs (0.1% hematocrit in PBS and 0.5 mL) were treated with increasing doses of *Hh*v (0–10 µg/mL) for 1 h at 37 °C. The cells were seeded in a 24-well culture plate on 12 mm sterile round coverslips. After treatment, the cells were washed with PBS and fixed with 2.5% glutaraldehyde solution for 2 h at RT. Further, samples were washed with three changes of PBS and dehydrated with a graded series of alcohol (50–100%) for 10 min each. The coverslips were dried in a desiccator for 12 h at RT. Then, samples were covered with a thin layer of gold (20 nm for 5 min) and observed under an SEM instrument using 3K× magnification (Carl Zeiss Ultra 55 FESEM, ZEISS, Germany) [38]. For inhibition studies, the *Hh*v (10 µg/mL) was independently pre-incubated with various doses (0–1000 µg/mL) of anti-venoms (Bharat, VINS, and *Hh*AV) and NAC (0–500 µM) for 10 min at RT before the treatment. 

### 2.21. Statistical Analysis

All the experiments presented in this study were carried out in triplicate and represented as a mean ± standard error of the mean (SEM). Statistical significance was determined using ordinary one-way analysis of variance (ANOVA), followed by Tukey’s multiple comparisons tests. Significance was accepted at a 95% confidence interval *p* > 0.05 (ns: non-significant), *p* < 0.05 (*), *p* < 0.01 (**), *p* < 0.001 (***), and *p* < 0.0001 (****). ‘*’ significant compared to control. Data were analyzed using the statistical package GraphPad Prism (GraphPad Software 8.0, San Diego, CA, USA). Confidence intervals (95%) to the effective dose (ED) of anti-venoms (Bharat, VINS, and *Hh*AV) and NAC were calculated using Microsoft Excel (Ver. 2021, Microsoft Corporation, 1 Microsoft Way, Redmond, WA, USA).

## 3. Results and Discussion

### 3.1. Hhv-Induced Crenation and Aggregation of RBCs

**Figure 1:**
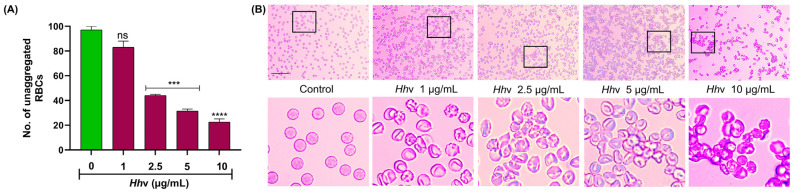
***Hh*v-induced crenation and aggregation of RBCs.** (**A**) Semi-quantitative estimation of RBCs aggregation. Washed RBCs were treated with increasing doses of *Hh*v (0–10 µg/mL) for 1 h at 37 °C. Free unaggregated RBCs remaining are estimated in 2–3 different fields of view. (**B**) Corresponding brightfield microscopic images of cells stained using Giemsa-stain. PBS-treated cells served as a negative control. After treatment, cells were stained using Giemsa stain and observed under the microscope using 40× magnification. The area enclosed by the black box in the top panel is magnified and shown below. Scale bar 30 µm. The data were presented as mean ± SEM (n = 3) and analyzed using ordinary one-way ANOVA followed by Tukey’s multiple comparisons test, ‘****’ *p* < 0.0001, ‘***’ *p* < 0.001, and ns (non-significant) > 0.05 compared to the PBS-treated cells.

Viper venoms induce fatal systemic coagulopathy and hemorrhaging, resulting in impaired blood circulation, thus severely affecting the rheological properties of the blood. However, in addition, the venom also causes massive edemas, hemorrhages, and tissue destruction at the site of the bite. Generally, indirect hemolytic activity is associated with all snake venoms including *Hh*v due to their PLA_2_ activity [39,40]. The lysophospholipids and free fatty acids generated due to membrane phospholipid hydrolysis exhibit detergent-like properties leading to the lysis of cells [41]. Nevertheless, *Hh*v did not show direct hemolysis [10]. The direct hemolytic activity is largely associated with the cytotoxins/cardiotoxins of the 3FTx toxins family of snake venoms. The cytotoxins/cardiotoxins directly perturb and destabilize the structural integrity of the plasma membrane resulting in lysis [42,43,44]. The lack of direct hemolytic activity is likely due to the absence of direct lytic factors/cytotoxins/cardiotoxins in this venom. Further, we investigated the venom-induced structural and morphological disturbances of human RBCs where it readily induced the aggregation of RBCs, and the effect was dose-dependent (Figure 1A). Interestingly, the venom exhibited a biphasic effect at lower doses (1–3 µg/mL) with an incubation period of 1 h, and the RBCs lost their natural discoid shape with conspicuous spikes (Crenation/serration) and blebbing of the plasma membrane. However, at higher doses (4–10 µg/mL), they underwent massive aggregation, as revealed by bright field microscopy (Figure 1B). The aggregation was irreversible, as phytochemicals and anti-venoms did not reverse the process (please see below for details). Crenation generally arises due to the loss of water/dehydration, resulting in loss of turgor pressure [45]. Thus, in response to venom action, RBCs lose their ideal shape and structure. This altered shape reduces their flexibility, making it difficult for them to smoothly squeeze through fine capillaries. Further, crenation would also impair their oxygen-transporting ability. Thus, this study provides an important lead for the possible venom-induced imbalance in the osmoregulatory functions of RBCs. Lectin-like toxins from *Trimeresurus mucrosquamatus* venom were reported to induce aggregation of RBCs [46]. In the recent past, *Hh*v was also reported to contain approximately 5.5 percent of C-type lectin-like proteins [47], as well as disintegrins (DIS), cysteine-rich secretory proteins (CRISP), and nerve growth factor (NGF) [48]. Snake venoms have been well-studied for their lectin-like molecules, popularly called CTL (C-type lectin-like) molecules, and these CTLs are also called snaclecs (snake C-type lectins) [49]. Snaclecs are heterodimers, having carbohydrate-recognition domain-related lectin-like domains that are incapable of binding sugars [50]. These readily interfere with platelet functions and strongly affect hemostasis by activating or inhibiting a wide range of plasma components [51,52]. However, Alborhagin (P-III SVMP) isolated from *Trimeresurus albolabris* venom is also known to interfere with platelet function [53]. Thus, the observed *Hh*v-induced RBC aggregation could be attributed to the lectin-like toxins in it. 

### 3.2. Hhv-Induced RBCs Aggregation Which Was Independent of ROS and Intracellular Calcium Ion Concentration 

**Figure 2:**
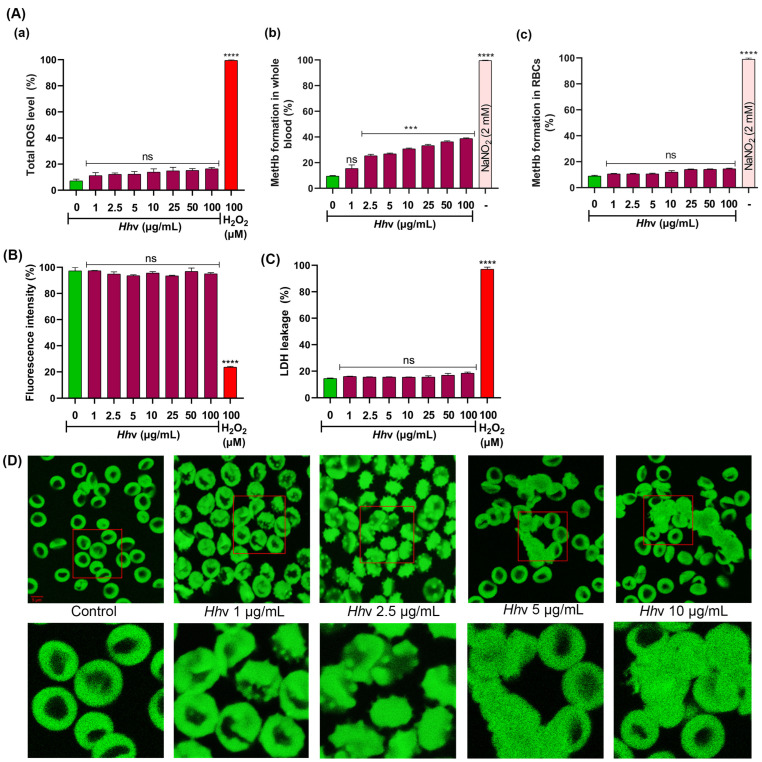
***Hh*v-induced methemoglobin formation and aggregations of RBCs.** (**A**(**a**)) Estimation of total ROS levels using DCFDA dye, (**A**(**b**)) estimation of methemoglobin (MetHb) formation in washed RBCs, and (**A**(**c**)) estimation of MetHb formation in whole blood. (**B**) Assessment of cell viability using calcein-AM fluorescence. (**C**) Estimation of LDH leakage in washed RBCs. In all the cases, the washed RBCs were independently treated with increasing doses of *Hh*v (0–100 µg/mL) for 1 h at 37 °C. For the MetHb formation assay, both washed RBCs and whole blood were independently treated and incubated for 16 h at 37 °C. For ROS, LDH leakage, and calcein AM assays, H_2_O_2_-treated (100 µM) cells served as a positive control. For the MetHb assay, NaNO_2_ (2 mM) served as a positive control. In all the cases, PBS-treated cells served as negative control. (**D**) Confocal microscopic images of washed RBCs loaded with calcein-AM fluorescent dye. Washed RBCs were independently treated with increasing doses of *Hh*v (0–10 µg/mL) for 1 h at 37 °C. PBS-treated cells served as the negative control. After treatment, cells were stained using calcein-AM dye and observed under a confocal microscope using 63× oil magnification (Carl Zeiss confocal microscope LSM 710, ZEISS, Germany). The area enclosed by the red box in the top panel is magnified and shown below. Scale bar 5 µm. The data were presented as mean ± SEM (n = 3) and analyzed using ordinary one-way ANOVA followed by Tukey’s multiple comparisons test, ‘****’ *p* < 0.0001, ‘***’ *p* < 0.001, and ns (non-significant) > 0.05 compared to the PBS-treated cells.

To obtain insight into the mechanism of *Hh*v-induced RBC aggregation, the generation of ROS and intracellular calcium ion concentrations were evaluated. Interestingly, the venom did not induce the generation of ROS in washed RBCs when incubated with increasing doses, 1 to 100 µg/mL for 1 h (Figure 2A(a)). Incidentally, the venom did not induce methemoglobin (MetHb) formation in washed RBCs (Figure 2A(b)) but showed significantly in whole blood (Figure 2A(c)). RBCs are essentially a bag full of hemoglobins, which are the oxygen carriers occupying over 95% of their volume, where MetHb formation occurs as a result of the oxidation of ferrous iron (Fe^2+^) to ferric iron (Fe^3+^). Characteristically, MetHb loses its ability to bind and transport oxygen [54]. In the whole blood, the venom readily stimulates neutrophils and other white blood cells to produce ROS by activating the NADPH-oxidase (NOX) enzyme [55]. The superoxide radical by dismutation becomes hydrogen peroxide (H_2_O_2_), which can easily diffuse and cross the plasma membrane barrier to reach neighboring RBCs and cause MetHb formation. Further, as expected, the venom did not increase the intracellular calcium ion concentration in washed RBCs. Generally, an increased ROS level is associated with increased intracellular Ca^2+^ concentration, followed by the externalization of membrane phosphatidylserine (PS), which is the hallmark signal for eryptosis [56] or apoptosis [57]. In this study, the venom did not cause any increase in ROS levels and intracellular calcium ion concentration, and no PS externalization was observed in washed RBCs (Figure S1), suggesting the non-cytotoxic mode of RBC aggregation. This was further supported by the calcein-AM cell death and LDH leakage assays where the cells revealed intense fluorescence and, further, there was no sign of LDH activity in the assay medium (Figure 2B,C). It is interesting that *Hh*v did not cause the death of RBCs but caused massive morphological abnormalities, as evidenced by bright field microscopic (Figure 1B) and calcein-AM fluorescence images (Figure 2D). Under lower doses (1–2.5 µg/mL), the venom-induced massive serrations where the plasma membrane exhibited dense protrusions, while, at higher doses (5–10 µg/mL), intense aggregation leading to clumping of RBCs was observed (Figure 2D). However, previously reported studies indicated potent cytotoxicity of *Hh*v to renal tubular cells (MDCK and LLC-MK2) [58] and cultured rat aorta smooth muscle cells [59]. Thus, *Hh*v appeared to exhibit cell or tissue-specific cytotoxicity. 

### 3.3. Hhv-Induced F-Actin Disarray and Deformability of RBCs 

**Figure 3:**
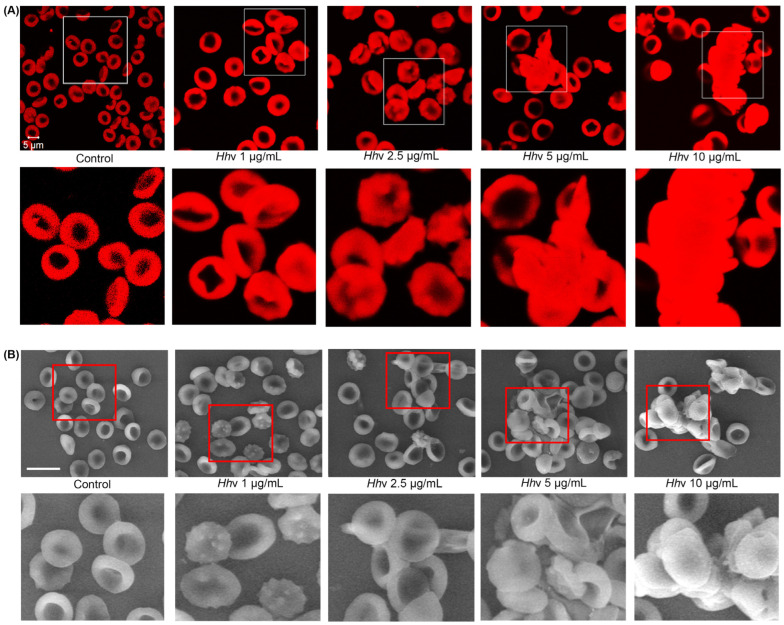
***Hh*v-induced F-actin disarray and deformability of RBCs.** (**A**) Confocal microscopic images of *Hh*v-treated RBCs stained using F-actin-specific phalloidin coupled with Alexa Fluor 488. The washed RBCs were independently treated with increasing doses of *Hh*v (0–10 µg/mL) for 1 h at 37 °C. PBS-treated cells served as a negative control. After treatment, cells were stained with F-actin-specific phalloidin-coupled Alexa Fluor 488 and observed under confocal microscopy using 63× oil magnification (Carl Zeiss confocal microscope LSM 710, ZEISS, Germany). (**B**) Scanning electron microscopic (SEM) images; washed RBCs were independently treated with increasing doses of *Hh*v (0–10 µg/mL) for 1 h at 37 °C. Samples were covered with a thin layer of gold (20 nm for 5 min) and observed under SEM using 3 K× magnification (Carl Zeiss Ultra 55 FESEM, ZEISS, Germany). The area enclosed by the white and red boxes in the top panel is magnified and shown below. Scale bars represent 5 µm for confocal and 3 µm for SEM images. The data are presented as mean ± SEM (n = 3).

Reversible deformability and fluidity are the special properties of RBCs that enable them to navigate evenly through the fine blood capillaries [60]. Cytoskeletal network connectivity involving spectrin, actin, protein 4.1R, ankyrin, actin-associated proteins, and band 3 protein is not a fixed structure [61]. There exists an extensive reversible dynamic remodeling of the RBC cytoskeleton called deformability [62]. Examining the effect of *Hh*v on the shape and deformability of RBCs, particularly the effect on the dynamics of actin, attracts special interest, as any change would severely affect the rheological property of RBCs in the blood. The cytochemical study of F-actin staining with Alexa Fluor 488 coupled phalloidin of *Hh*v-treated RBCs displayed a uniformly distributed deep red color in confocal images as compared to the intense red color being concentrated in the edges of control RBCs, revealing a ring-like appearance. The varied distribution pattern of red color suggests the stress-induced F-actin cytoskeletal disarray and shape transformation of RBCs from biconcave to biconvex form. This suggests venom-induced cytoskeletal deformation. Under lower doses (1–2.5 µg/mL), the venom induces shape change, while at higher doses (5–10 µg/mL), massive aggregation of RBCs was manifested by confocal microscopy (Figure 3A). Many Elapidae snake venoms were reported to cause the formation of stomatocytes in the initial time of exposure, with unusual morphological protrusions and F-actin cytoskeletal aggregations; however, they caused hemolysis of RBCs upon prolonged exposure [63]. Further, the scanning electron microscopy (SEM) study provides strong evidence for our observations and confirms the *Hh*v-induced major structural abnormalities as well as aggregation of washed RBCs (Figure 3B). South African puff adder (*Bitis arietans*) snake venom was reported to induce an eryptotic type of cell death in RBCs [64]. Eryptosis is characterized by the shrinkage of cells, surface blebbing, and scrambling of plasma membrane phospholipids [65]. Therefore, it is likely that *Hh*v-induced aggregation of RBCs follows an unknown mechanism, which needs further study.

### 3.4. Hhv-Induced RBC Aggregates Exhibit Pro-Coagulant Activity but Did Not Affect Platelets

**Figure 4:**
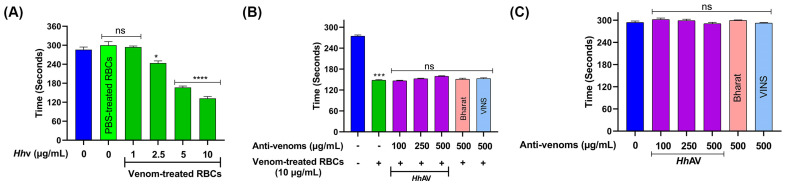
**Pro-coagulant activity of the *Hh*v-treated RBC aggregates.** (**A**) RBCs were independently treated with increasing doses of *Hh*v (0–10 µg/mL) for 1 h at 37 °C. The respective RBC aggregates obtained were washed thoroughly with PBS and tested for their effect on plasma recalcification time. The assay mixture contains 0.2 mL platelet-poor plasma (PPP) and respective RBC aggregates, pre-incubated for 1 min at 37 °C, and clotting was initiated by adding 0.25 M CaCl_2_. (**B**) Effect of anti-venoms on aggregated RBCs induced pro-coagulant activity. The pro-coagulant RBC aggregates obtained at 10 µg/mL venom were washed with PBS and used. RBC aggregates (20 µL) were pre-incubated with different doses (0–500 µg/mL) of anti-venoms (Bharat, VINS, and *Hh*AV) for 5 min at RT, this mixture was then added to 0.2 mL PPP and incubated for 1 min at RT. (**C**) Effect of anti-venoms on plasma recalcification time. PPP, 0.2 mL was incubated with different doses (0–500 µg/mL) of anti-venoms (Bharat, VINS, and *Hh*AV) for 5 min at RT. The plasma re-calcification time was determined by adding 0.25 M CaCl_2_. In all the cases, clotting time was recorded in seconds against a light source. The data are presented as mean ± SEM (n = 3) and analyzed using ordinary one-way ANOVA followed by Tukey’s multiple comparisons test, ‘****’ *p* < 0.0001, ‘***’ *p<* 0.001, ‘*’ *p* < 0.05, and ns (non-significant) > 0.05 compared to the PBS-treated cells.

The *Hh*v-induced washed RBC aggregates when tested, readily caused the coagulation of human citrated PPP, where it significantly decreased coagulation time. Thus, the *Hh*v-induced RBC aggregates exhibit pro-coagulant activity. Interestingly, the observed pro-coagulant activity was proportional to the RBC aggregates obtained with respective doses of *Hh*v (Figure 4A). Remarkably, the pro-coagulant activity of the RBC aggregates was not affected by anti-venoms. Both *Hh*AV and therapeutic polyvalent anti-venoms did not inhibit the pro-coagulant activity of RBC aggregates (Figure 4B). The anti-venoms (Bharat, VINS, and *Hh*AV) alone did not affect the plasma recalcification time (Figure 4C). Envenomation by both Elapidae and Viperidae snakes is known to cause VICC [19,40]. Snake venoms are the depot of various coagulation factors such as prothrombin activators, factor V and factor X activators, thrombin-like enzymes or fibrinogenases, fibrinolytic enzymes, and other factors readily degrade/consume their respective substrates, leading to VICC [66,67,68]. The *Hh*v-induced RBC aggregates by exhibiting pro-coagulant activity readily complement VICC and thus critically affect blood circulation. The serrated and deformability of RBCs were found to induce less platelet aggregation as compared to healthy RBCs [69]. Thus, as expected, the venom-induced crenated RBC aggregates did not induce platelet aggregation in both PRP and washed platelets (Figure S2). 

### 3.5. Hhv-Induced Alterations in Membrane Fluidity and Metabolic Functions in Washed RBCs 

**Figure 5:**
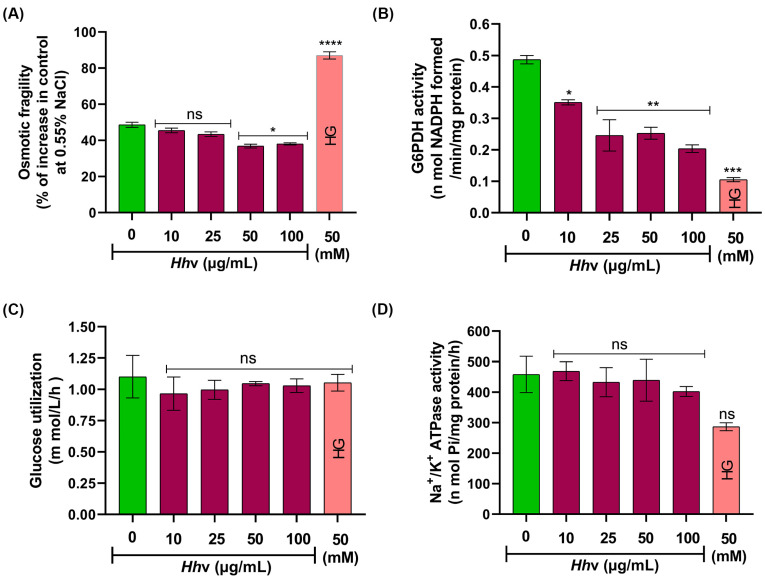
**Effect of *Hh*v on membrane integrity and metabolic functions of RBCs.** (**A**) Measurement of osmotic fragility. (**B**) Determination of G6PDH activity. (**C**) Estimation of glucose utilization in RBCs by GOD-POD method. (**D**) Estimation of Na^+^/K^+^ -ATPase activity in RBCs membrane. In all the cases, the washed RBCs were independently treated with increasing doses of *Hh*v (0–100 µg/mL) for 1 h at 37 °C. The PBS-treated samples served as negative control. High glucose (HG) 50 mM served as a positive control. The data are presented as mean ± SEM (n = 3) and analyzed using ordinary one-way ANOVA followed by Tukey’s multiple comparisons test, ‘****’ *p* < 0.0001, ‘***’ *p* < 0.001, ‘**’ *p* < 0.01, ‘*’ *p* < 0.05 and ns (non-significant) > 0.05 compared to the PBS-treated cells.

*Hh*v readily affects the viscosity of the plasma membrane of washed RBCs. Upon *Hh*v treatment, RBCs were less susceptible to hemolysis, and the effect was dose-dependent. The observed resistance to hemolysis suggests the venom-induced stiffness of the RBC plasma membrane (Figure 5A). Generally, RBCs are susceptible to easy hemolysis during oxidative stress due to increased osmotic fragility [70]. *Hh*v is likely to affect phospholipid packing patterns, as well as the amount of unsaturated fatty acids and cholesterol, as they are the key factors that regulate the extent of the fluidity of the plasma membrane [71]. The venom dose-dependently inhibited glucose 6 phosphate dehydrogenase enzyme activity (Figure 5B). Being the principal generator of NADPH, largely, glucose-6 phosphate dehydrogenase protects the RBCs from oxidative stress, as its deficiency results in acute hemolytic anemia [72]. However, it is interesting that in our study, hemolysis and glucose 6 phosphate dehydrogenase activity are nonreciprocally related. RBCs, despite binding and transporting oxygen to various parts of the body, themselves lack efficient oxygen-utilizing and energy-generating centers, the mitochondria. Thus, the metabolic activities are propeled solely by the energy synthesized from the Embden–Meyerhof–Parnas (EMP) pathway [73]. Excitingly, the venom did not affect either the uptake of glucose (Figure 5C) or Na^+^/K^+^ -ATPase activity (Figure 5D) of washed RBCs at concentrations as high as 100 µg/mL. Thus, based on the obtained results, *Hh*v could affect the functionality of RBCs.

### 3.6. Hhv-Induced RBCs Aggregation, Inhibition, or Dis-Aggregation by Bioactives, Sugars, and Anti-Venoms

**Figure 6:**
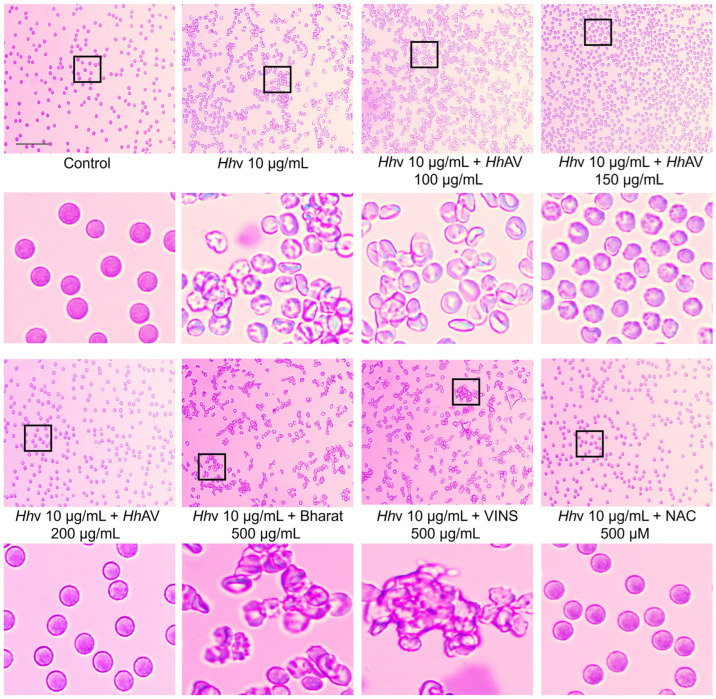
**Neutralization/non-neutralization efficacy of *Hh*v-induced RBCs aggregation by anti-venoms and NAC.** Bright-field microscopic images of cells stained using Giemsa stain. *Hh*v (10 µg/mL) was independently pre-treated with various doses (0–500 µg/mL) of anti-venoms (Bharat, VINS, and *Hh*AV) and NAC (0–500 µM) for 10 min at RT before treatment. These reaction mixtures were treated independently to washed RBCs and incubated for 1 h at 37 °C. PBS-treated cells served as a negative control. After treatment, cells were stained using Giemsa stain and observed under the microscope using 40× magnification. The area enclosed by the black box in the top panel is magnified and shown below, scale bar of 30 µm. The data are presented as mean ± SEM (n = 3).

Reversible RBC aggregation is a normal physiological event that happens in normal circulating blood during stasis or in low-flow circumstances [74]. *Hh*v readily induces stress in RBCs; as a result, they undergo aggregation. Anti-venom being the final remedial agent, it was important to establish the inhibition of aggregation of RBCs as well as the disaggregating potential of already formed RBC aggregates by the therapeutic polyvalent anti-venoms. Therapeutic polyvalent anti-venoms (Bharat and VINS), which were raised against the cocktail of venoms from the ‘Big-Four’ snakes of the Indian subcontinent, did not inhibit the aggregation of RBCs, nor could they disaggregate the RBCs aggregates even at doses as high as 1000 µg/mL (95% CL: 986.15 ± 201 µg/mL) in pre-incubation/co-treatment/post-treatment studies (Figure S3). However, in contrast, the affinity-purified rabbit monovalent anti-venom raised against *Hh*v could inhibit the aggregation of RBCs in pre-incubation and/or co-treatment studies but did not inhibit the already formed aggregates. In addition, pre-incubation or co-treatment of *Hh*AV effectively alleviated the crenatures or spikes of the plasma membrane and protected RBCs from retaining their natural biconcave shape (Figure 6) at a dose of 200 µg/mL (95% CL: 216.3 ± 22.19 µg/mL). Additionally, the confocal and SEM studies provided strong evidence for reversing the RBC aggregation by *Hh*AV (Figures S4–S6). It is very well documented that even the neutralizing therapeutic anti-venoms can only inhibit the toxic effects of snake venoms but cannot reverse the venom-induced toxic effects [75,76,77]. Paradoxically, as none of the anti-venoms cause disaggregation or reverse the already formed aggregates, except for *Hh*AV, our study strongly suggests the early administration of therapeutic anti-venom so as to neutralize the toxicity of free toxins before they bind their targets and exert irreversible toxicity [78]. 

Further, in the absence of a suitable anti-venom therapy for *Hh* envenomation, in an attempt to find a first-aid agent that could inhibit aggregation of RBCs or disaggregate RBC aggregates, we have investigated various bioactive molecules, such as melatonin, curcumin, fisetin, berberine, quercetin, and N-acetylcysteine (NAC) and sugars such as mannose and galactose. Mannose and galactose were used for the inhibition study as galactose and mannose-specific lectins have been reported from different snake venoms [79,80]. In both pre-incubation and co-treatment studies, none of the bioactives and sugars tested inhibited aggregation of RBCs or disaggregated the RBC aggregates (Figure S7), except for NAC, where NAC dose-dependently inhibited the aggregation; however, this also failed to disaggregate the RBC aggregates. At 500 µM (95% CL: 503 ± 21.5 µM), concentrations of NAC efficiently inhibited the aggregation process (Figure S8A) but failed to disaggregate the RBC aggregates, even at doses as high as 1000 µM (95% CL: 865 ± 103.72 µM) (Figure S8B). Thus, NAC appears highly promising as a first aid agent, at least to protect RBCs from undergoing likely fatal aggregates. The aggregates may float as an embolus and may lodge in fine capillaries of vital organs such as kidneys, the heart, and the brain, leading to ischemia and causing further complications [81,82]. Thus, a likely fatal event may be effectively managed by the timely administering of NAC as a first-aid agent. 

## 4. Conclusions

The present study elucidates the hemotoxic property of *Hh*v and has contributed valuable insights into its pathophysiological impacts on RBCs. Similar to sickle cell anemia, venom distorts the morphology of RBCs, minimizing their capacity for mobility and thereby rendering it more complicated for them to circulate through minuscule capillaries as well as impacting their ability to transport oxygen [83]. This probably leads to a blockage of delicate capillaries of vital organs including the brain, heart, and kidneys. This not only causes excruciating discomfort but may also damage the organs. Further, this study systematically unravels the ineffectiveness of bioactives, sugars, and therapeutic polyvalent anti-venoms (except for *Hh*AV and NAC) against the venom-induced aggregation of RBCs. Thus, prevention of venom-induced morphological and functional anomalies and irreversible aggregation of RBCs remains a significant challenge and may be achieved by early administration of *Hh*AV or administration of NAC as a potential first-aid agent, particularly during a forced delay during the treatment process. Nevertheless, the venom’s unique ability to induce morphological changes and persistent aggregation of RBCs, regardless of reactive oxygen species and intracellular Ca^2+^ concentration (but without exerting the death of RBCs), underscores the necessity for further research into its complex mechanisms of action.

## Data Availability

Data are contained within the article and Supplementary Materials.

## Supplementary Materials

The following supporting information can be downloaded at: https://www.mdpi.com/article/10.3390/cells13120994/s1, Figure S1: Determination of eryptosis markers in washed RBCs treated with *Hh*v. Figure S2: Effect of RBC aggregates on platelet aggregation. Figure S3: Effect of therapeutic polyvalent anti-venoms against *Hh*v-induced RBCs aggregations. Figure S4: Neutralization/non-neutralization efficacy of *Hh*v-induced RBCs aggregation by anti-venoms and NAC. Figure S5: Neutralization/non-neutralization efficacy of *Hh*v-induced RBCs aggregation by anti-venoms and NAC. Figure S6: Neutralization/non-neutralization efficacy of *Hh*v-induced RBCs aggregation by anti-venoms and NAC. Figure S7: Effect of bioactives and sugars against *Hh*v-induced RBCs aggregation. Figure S8: Neutralization/non-neutralization efficacy of NAC against *Hh*v-induced RBCs aggregations.
