# Supplementary material for: Hump-Nosed Pit Viper (Hypnale hypnale) Venom-Induced Irreversible Red Blood Cell Aggregation, Inhibition by Monovalent Anti-Venom and N-Acetylcysteine
Source: Cells. 2024 Jun 7;13(12):994. doi: 10.3390/cells13120994 (PMC11201549; doi:10.3390/cells13120994)
Supplement: Supplementary file 1 [file cells-13-00994-s001.zip › cells-2997516-supplementary.pdf]

Hump-Nosed Pit Viper (Hypnale hypnale) Venom-Induced Irreversible Red Blood Cell Aggregation, Inhibition by Monovalent Anti-Venom and N-Acetylcysteine

Vaddaragudisalu D. Sandesha [1], Puttaswamy Naveen [1], Kurnegala Manikanta [1], Shanmuga S. Mahalingam [2], Kesturu S. Girish [3,*] and Kempaiah Kemparaju [1,*]

1 Department of Studies in Biochemistry, University of Mysore, Manasagangotri, Mysuru 570006, Karnataka, India

2 Department of Biological Sciences, School of Dental Medicine, Case Western Reserve University, Cleveland, OH 44106, USA

3 Department of Studies and Research in Biochemistry, Tumkur University, Tumakuru 572103, Karnataka, India

*** Corresponding authors:** Kempaiah Kemparaju (kemparajuom@gmail.com)

Kesturu S. Girish (ksgbaboo@gmail.com)

Supporting information

Figure S1: Determination of eryptosis markers in washed RBCs treated with *Hhv*. (A) Estimation of intracellular Ca2+ ion concentration in RBCs. (B) Determination of PS externalization in RBCs. In both cases, washed RBCs were independently treated with increasing doses of *Hhv* (0–100 µg/mL) for 1 h at 37 °C. To detect the intracellular Ca2+ ions, the cells were stained using Fura-2 AM, and fluorescent intensity was measured using a Tecan multi-mode plate reader (Infinite 200 Pro, Tecan Grodig, Austria). To detect the PS externalization, the cells were stained using annexin-V FITC and analyzed by FACS Verse flow cytometer (BD Biosciences, San Jose, CA, USA). In both cases, H2O2-treated (100 µM) cells served as a positive control and PBS-treated cells served as a negative control. The data is presented as mean ± SEM (n=3) and analyzed using ordinary one-way ANOVA followed by Tukey's multiple comparisons tests, '**** p <0.0001 and ns (non-significant) >0.05. '*' significant compared to the PBS-treated cells.

Figure S2: Effect of RBC aggregates on platelet aggregation. (A) Effect of aggregated RBCs on the aggregation of PRP. (B) Effect of aggregated RBCs on the aggregation of washed platelets. In both cases, the aggregation was monitored by adding 20 µL of aggregated RBCs using a Chronolog dual-channel whole blood/optical Lumi aggregation system (Model-700) with constant stirring at 1200 rpm for 6 min. Thrombin 0.1U was used as a positive control. The data is presented as mean ± SEM (n=3).

Figure S3: Effect of therapeutic polyvalent anti-venoms against *Hhv*-induced RBCs aggregations. Bright-field microscopic images of cells stained using Giemsa stain. *Hhv* (10 µg/mL) was independently preincubated with various doses (0-1000 µg/mL) of therapeutic polyvalent anti-venoms (A) Bharat and (B) VINS for 10 min at RT before treatment. This respective assay mixture was independently treated with washed RBCs and incubated for 1h at 37 °C. PBS-treated cells served as a negative control. After treatment, cells were stained using Giemsa stain and observed under the microscope using 40× magnification. The area enclosed by the black box in the top panel is magnified and shown below. Scale bar 30 µm. The data is presented as mean ± SEM (n=3).

Figure S4: Neutralization/non-neutralization efficacy of *Hhv*-induced RBCs aggregation by an-ti-venoms and NAC. Confocal microscopic images of washed RBCs using calcein-AM fluorescent dye. *Hhv* (10 µg/mL) was independently pretreated with various doses (0-1000 µg /mL) of an-ti-venoms (Bharat, VINS, and *HhAV*) and NAC (0-500 µM) for 10 min at RT before treatment. These reaction mixtures were treated independently to washed RBCs and incubated for 1 h at 37 °C. PBS-treated cells served as a negative control. After treatment,

cells were stained using calcein AM fluorescent dye and observed under a confocal microscope using 63× oil magnification (Carl Zeiss confocal microscope LSM 710, ZEISS, Germany). The area enclosed by the red box in the top panel is magnified and shown below. Scale bar 5 µm. The data is presented as mean ± SEM (n=3).

Figure S5: Neutralization/non-neutralization efficacy of *Hhv*-induced RBCs aggregation by an-ti-venoms and NAC. The confocal microscopic images of washed RBCs using F-actin-specific stain phalloidin coupled with Alexa Fluor 488. *Hhv* (10 µg/mL) was independently pretreated with various doses (0-1000 µg/mL) of anti-venoms (Bharat, VINS, and *HhAV*) and NAC (0-500 µM) for 10 min at RT before treatment. These reaction mixtures were treated independently to washed RBCs and incubated for 1 h at 37 °C. PBS-treated cells served as a negative control. After treatment, cells were stained with F-actin-specific phalloidin-coupled Alexa Fluor 488 and observed under the confocal microscope using 63× oil magnification (Carl Zeiss confocal microscope LSM 710, ZEISS, Germany). The area enclosed by the white box in the top panel is magnified and shown below. Scale bar 5 µm. The data is presented as mean ± SEM (n=3).

Figure S6: Neutralization/non-neutralization efficacy of *Hhv*-induced RBCs aggregation by an-ti-venoms and NAC. SEM images of RBCs, *Hhv* (10 µg/mL) was independently pretreated with various doses (0-1000 µg/mL) of anti-venoms (Bharat, VINS, and *HhAV*) and NAC (0-500 µM) for 10 min at RT before treatment. These reaction mixtures were treated independently to washed RBCs and incubated for 1 h at 37 °C. PBS-treated cells served as a negative control. After treatment, cells were fixed using 2.5 % glutaraldehyde and dehydrated with a graded series of alcohol (50–100%) for 10 min each. The coverslips were dried in a desiccator for 12 h at RT. Samples were covered with a thin layer of gold (20 nm for 5 min) and observed under SEM (Carl Zeiss Ultra 55 FESEM, ZEISS, Germany) using 3K× magnification. The area enclosed by the red box in the top panel is magnified and shown below. Scale bar 3 µm. The data is presented as mean ± SEM (n=3).

Figure S7: Effect of bioactives and sugars against *Hhv*-induced RBCs aggregation. Bright-field microscopic images of cells stained using Giemsa stain. *Hhv* (10 µg/mL) was independently preincubated with various doses (0-50 µM) of bioactives such as melatonin, curcumin, fisetin, berberine, and quercetin, and various doses (0-500 µg/mL) of sugars (Mannose and galactose) for 10 min at RT before treatment. This assay mixture was further treated with washed RBCs and incubated for 1 h at 37 °C. PBS-treated cells served as a negative control. After treatment, cells were stained using Giemsa stain and observed under

the microscope (40× magnification). The area enclosed by the black box on the top panel is magnified and shown below. Scale bar 30 µm. The data is presented as mean ± SEM (n=3).

Figure S8: Neutralization/non-neutralization efficacy of NAC against *Hhv*-induced RBCs aggregations. (A) Bright-field microscopic images of cells stained using Giemsa stain. *Hhv* (10 µg/mL) was independently preincubated with increasing doses of NAC (0-500 µM) for 10 min at RT before treatment. This reaction mixture was further treated with washed RBCs and incubated for 1 h at 37 °C. (B) Bright-field microscopic images of cells stained using Giemsa stain. The various doses of NAC (0-1000 µM) were independently treated along with *Hhv* (10 µg/mL) and incubated at 37 °C for 1 h. PBS-treated cells served as a negative control. After treatment, cells were stained using Giemsa stain and observed under the microscope (40× magnification). The area enclosed by the black box on the top panel is magnified and shown below. Scale bar 30 µm. The data is presented as mean ± SEM (n=3).

Figures

Figure S1

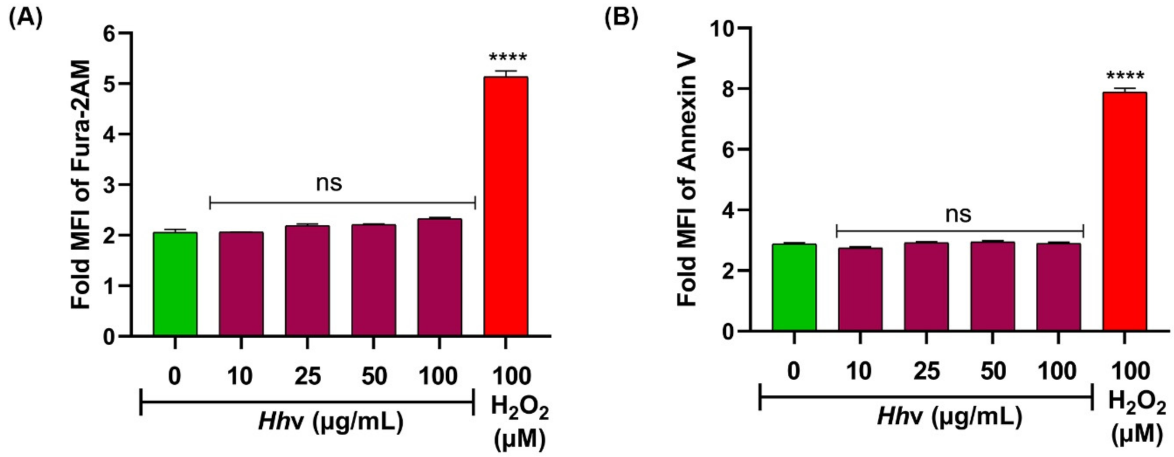

Figure S2

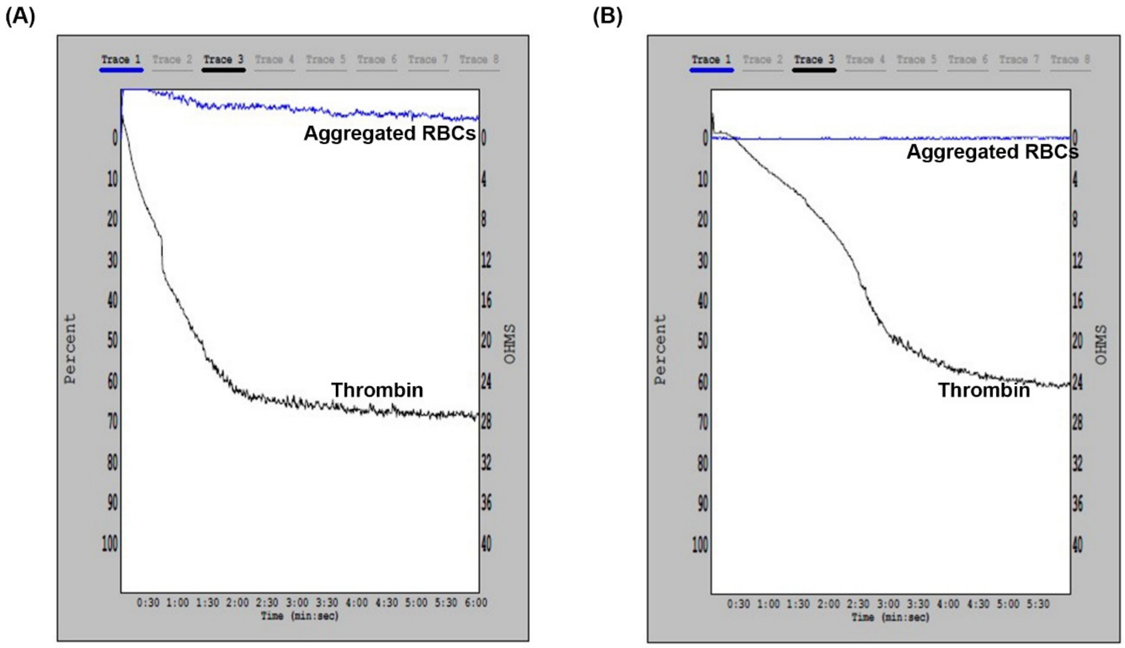

Figure S3

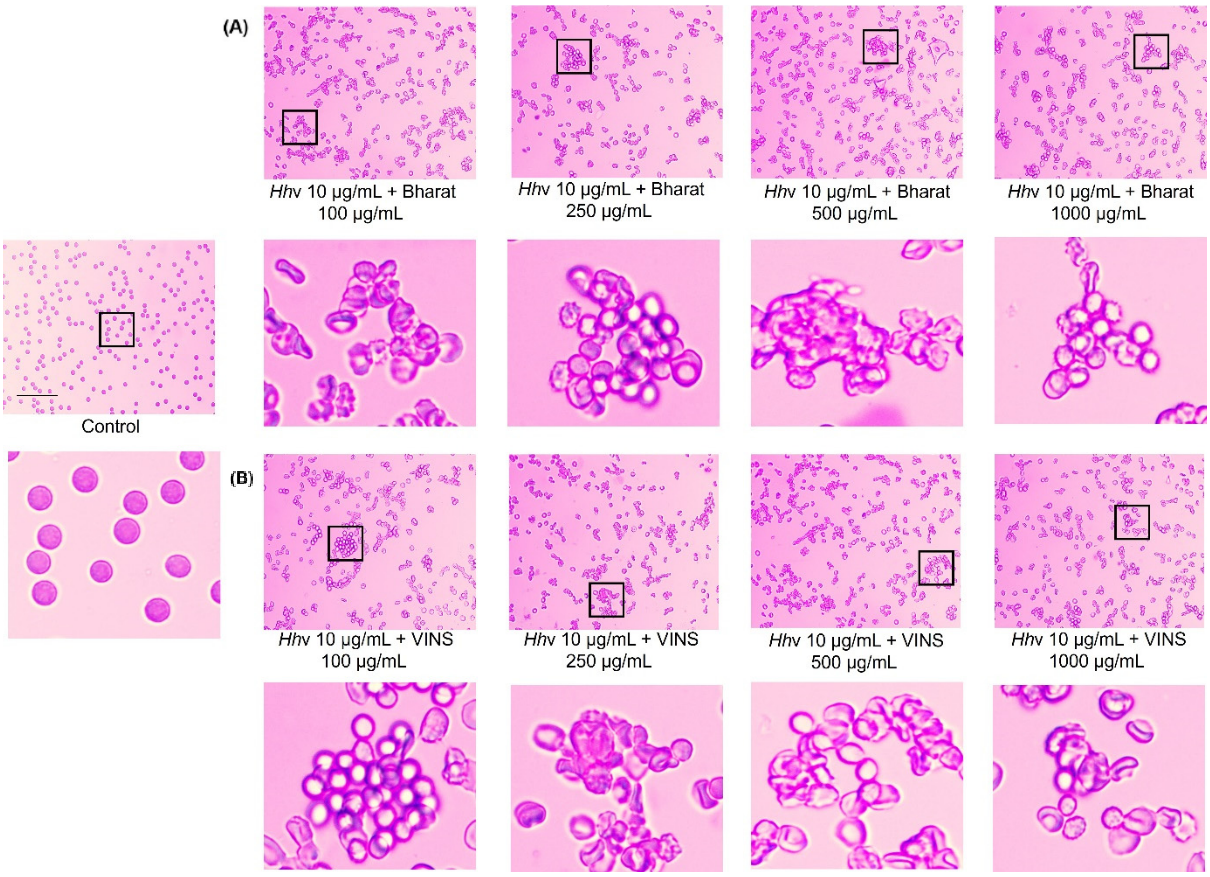

Figure S4

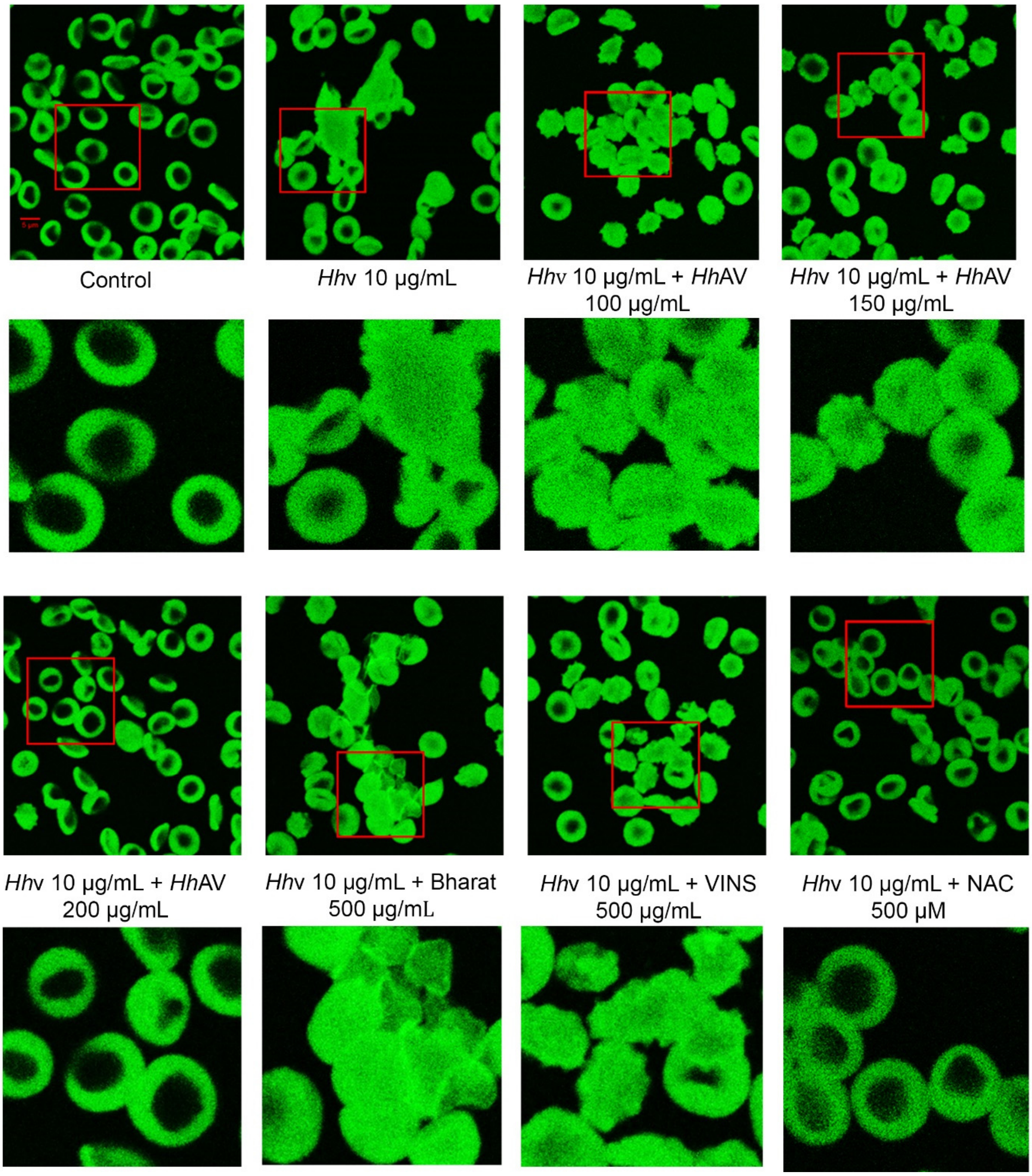
Control
*Hh*v 10 µg/mL
*Hh*v 10 µg/mL + *Hh*AV 100 µg/mL
*Hh*v 10 µg/mL + *Hh*AV 150 µg/mL
*Hh*v 10 µg/mL + *Hh*AV 200 µg/mL
*Hh*v 10 µg/mL + Bharat 500 µg/mL
*Hh*v 10 µg/mL + VINS 500 µg/mL
*Hh*v 10 µg/mL + NAC 500 µM

Figure S5

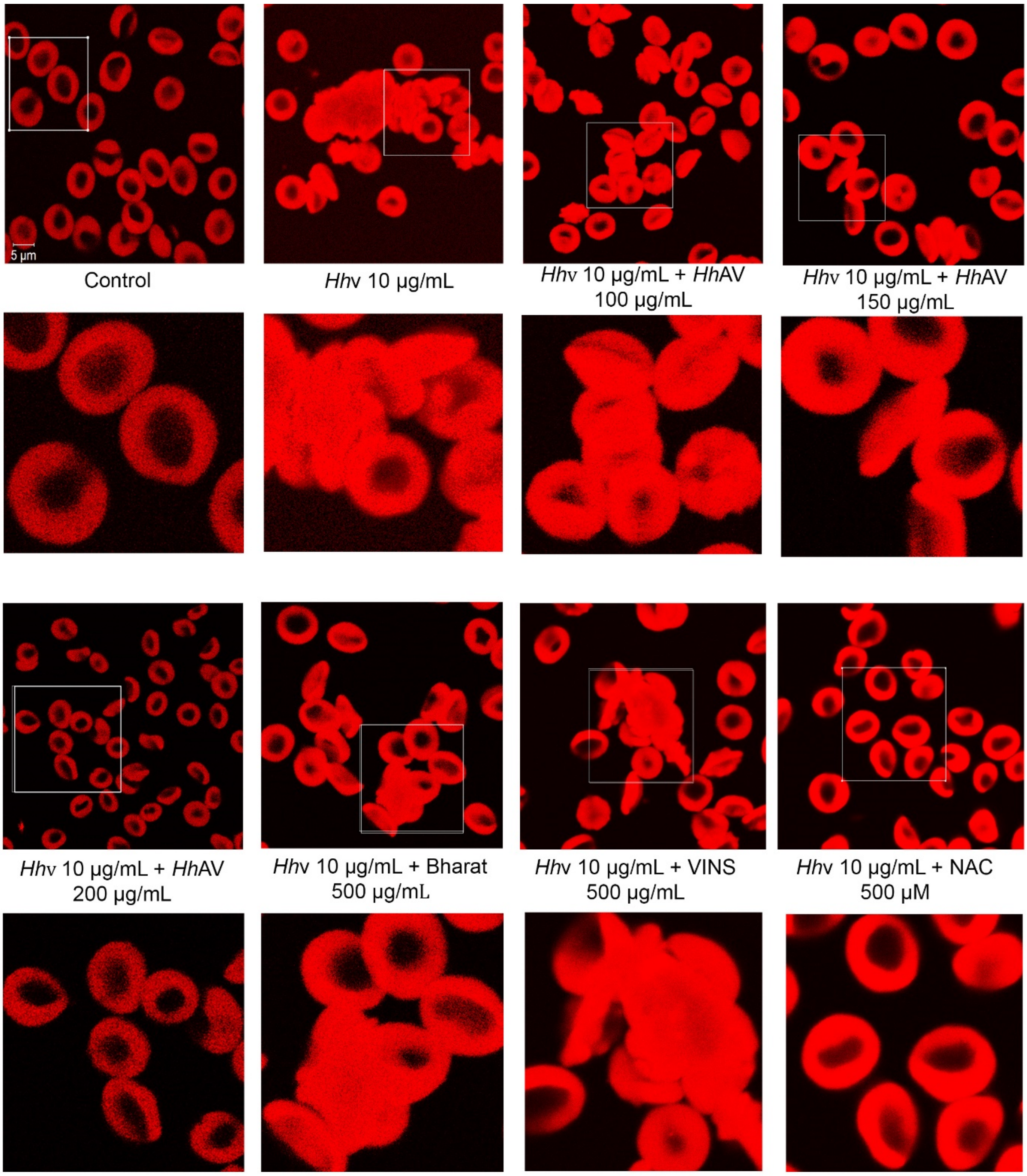
5 µm
Control
*Hh*v 10 µg/mL
*Hh*v 10 µg/mL + *Hh*AV 100 µg/mL
*Hh*v 10 µg/mL + *Hh*AV 150 µg/mL
*Hh*v 10 µg/mL + *Hh*AV 200 µg/mL
*Hh*v 10 µg/mL + Bharat 500 µg/mL
*Hh*v 10 µg/mL + VINS 500 µg/mL
*Hh*v 10 µg/mL + NAC 500 µM

Figure S6

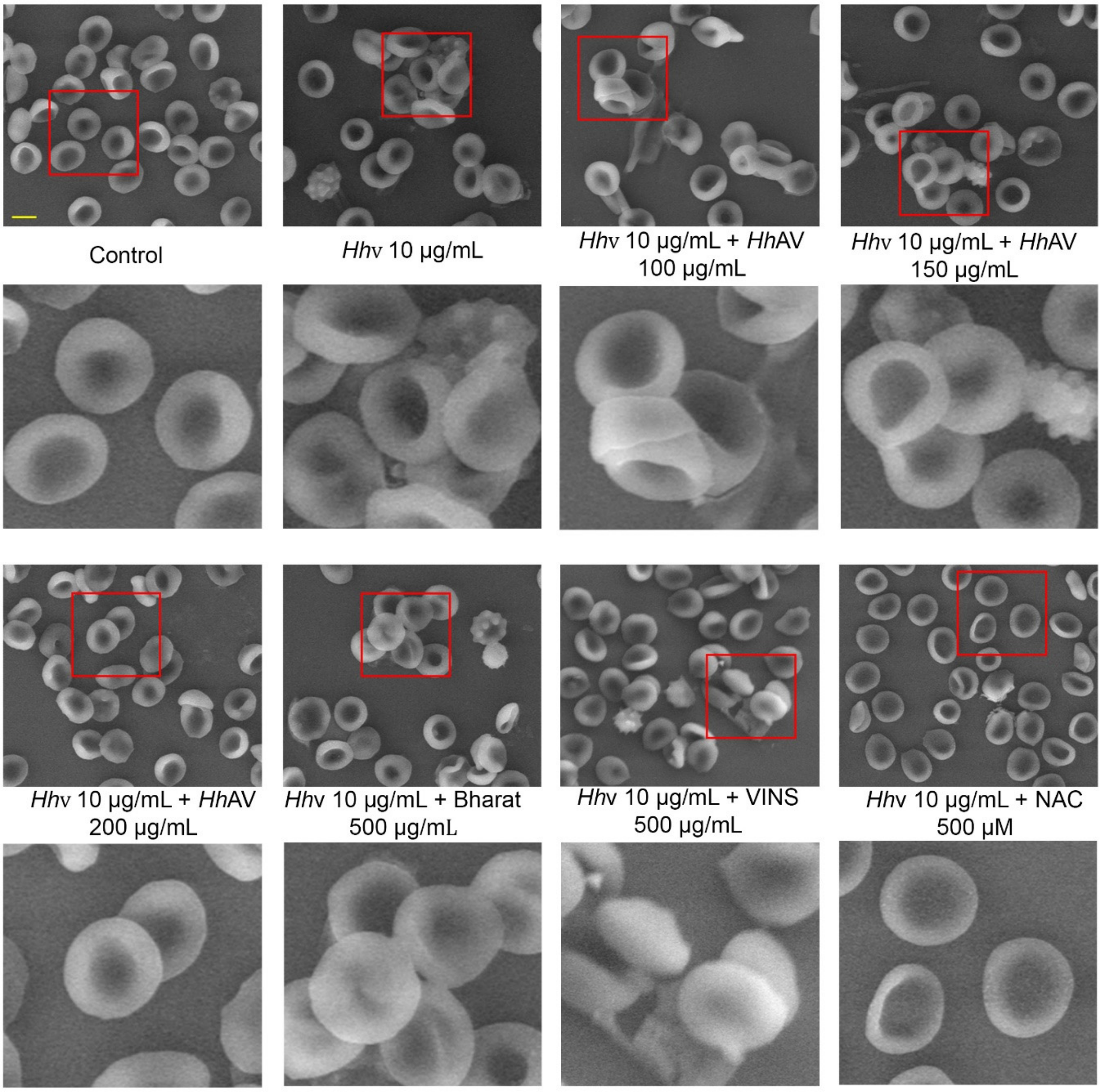
Control
*Hh*v 10 μg/mL
*Hh*v 10 μg/mL + *Hh*AV 100 μg/mL
*Hh*v 10 μg/mL + *Hh*AV 150 μg/mL
*Hh*v 10 μg/mL + *Hh*AV 200 μg/mL
*Hh*v 10 μg/mL + Bharat 500 μg/mL
*Hh*v 10 μg/mL + VINS 500 μg/mL
*Hh*v 10 μg/mL + NAC 500 μM

Figure S7

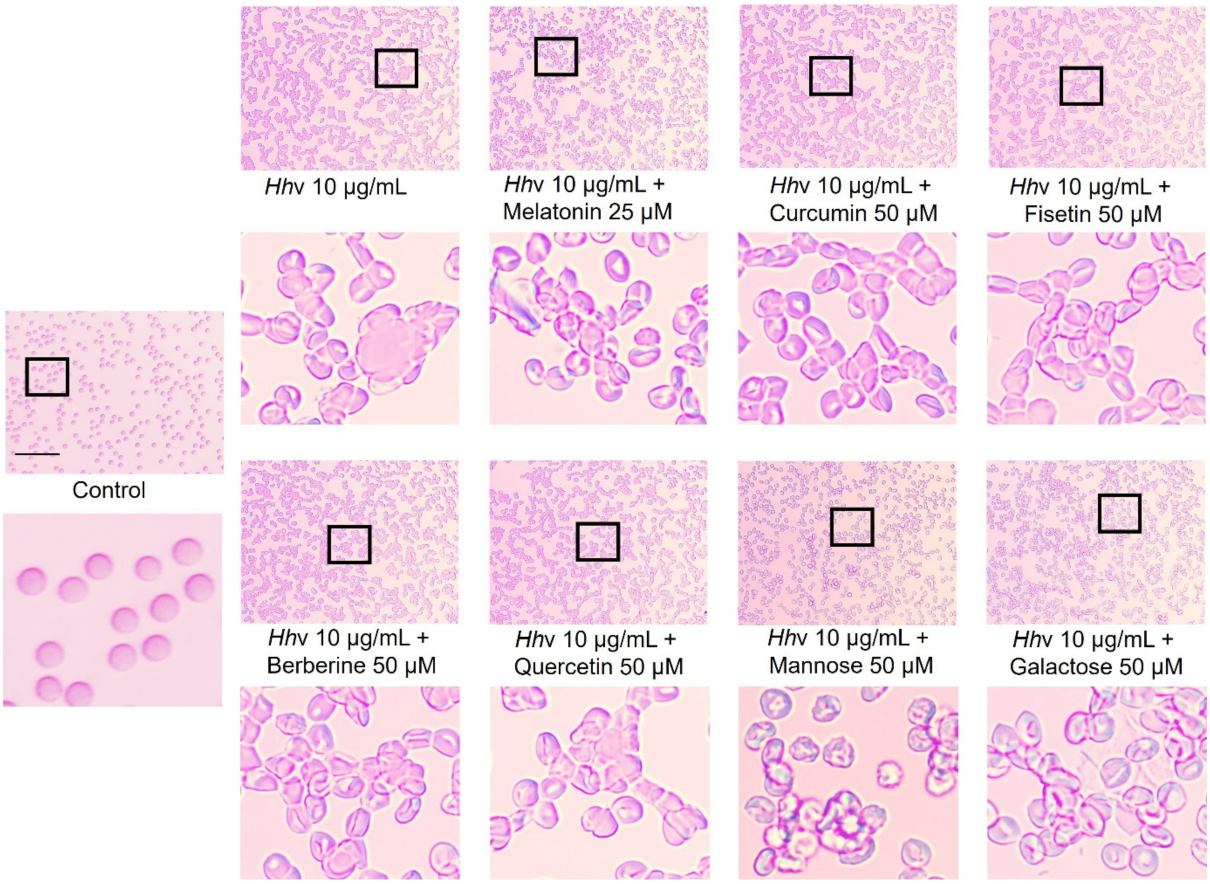
Control
Hhv 10 μg/mL
Hhv 10 μg/mL + Melatonin 25 μM
Hhv 10 μg/mL + Curcumin 50 μM
Hhv 10 μg/mL + Fisetin 50 μM
Hhv 10 μg/mL + Berberine 50 μM
Hhv 10 μg/mL + Quercetin 50 μM
Hhv 10 μg/mL + Mannose 50 μM
Hhv 10 μg/mL + Galactose 50 μM

Figure S8

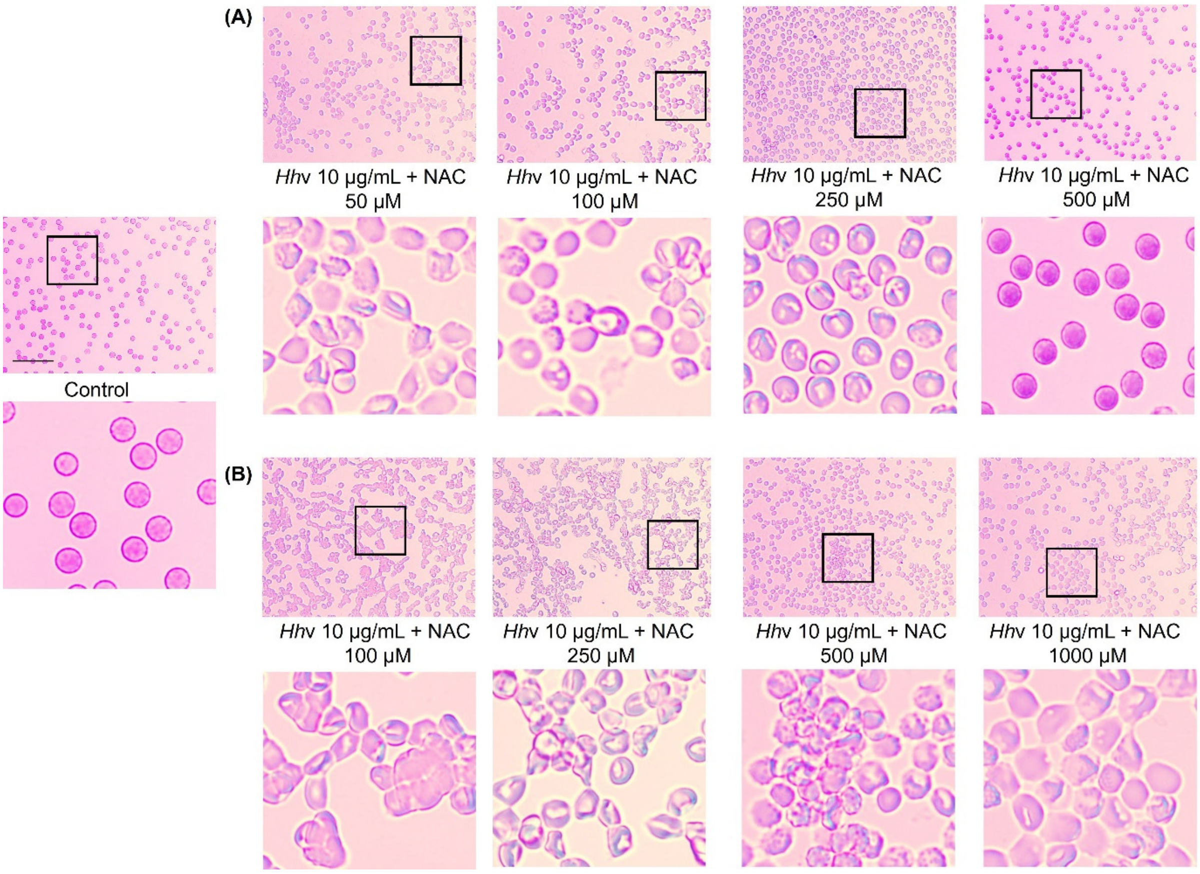
(A)
*Hh*v 10 µg/mL + NAC 50 µM
*Hh*v 10 µg/mL + NAC 100 µM
*Hh*v 10 µg/mL + NAC 250 µM
*Hh*v 10 µg/mL + NAC 500 µM
Control
(B)
*Hh*v 10 µg/mL + NAC 100 µM
*Hh*v 10 µg/mL + NAC 250 µM
*Hh*v 10 µg/mL + NAC 500 µM
*Hh*v 10 µg/mL + NAC 1000 µM
